# Reliability and validity of a self-developed virtual reality-based test battery for assessing motor skills in sports performance

**DOI:** 10.1038/s41598-025-89385-3

**Published:** 2025-02-20

**Authors:** Stefan Pastel, Florian Klenk, Dan Bürger, Florian Heilmann, Kerstin Witte

**Affiliations:** 1 Institute III: Sports Science, Department of Sports Engineering and Movement Science, Otto-von-Guericke-University, Magdeburg, Germany; 2 Institute for Sports Science, Movement Science Lab, Martin-Luther-University Halle-Wittenberg, Halle, Germany

**Keywords:** Virtual reality, Motor skills, Jump and reach, Reaction, Test battery, Virtual opponent, Scientific data, Human behaviour

## Abstract

Athletes must master various motor skills for success in their sports. To assess performance and identify areas of improvement, effective sports-motoric tests are essential. Key abilities such as reaction time, jumping, and complex movement coordination are critical. Virtual reality (VR) offers a practical, traditional equipment-free tool for assessment, though new VR-based tests must be evaluated first. We evaluated a self-developed test battery to measure reaction time (drop-bar test), jumping ability (jump and reach test), and parkour execution involving multiple complex motor tasks (with/without a virtual opponent). 32 participants completed these tests twice in real environment (RE) and VR (pre- and post-test). Intraclass correlation coefficients showed high reliability for reaction time in RE (0.858) and VR (0.888), with moderate significant correlations between them (*r* = .445), suggesting validity. The jump and reach test showed even better reliability (RE: 0.944, VR: 0.886) with strong correlations between RE and VR (*r* = .838). The parkour test showed lower reliability (x̄ 0.770), particularly for one task, with significant differences between the conditions indicating different behavior in VR. However, the addition of a virtual opponent eliminated these differences. VR appears to be a promising alternative to traditional testing methods, revealing comparable values across conditions.

## Introduction

Motoric skills and abilities encompass a wide range of attributes that athletes must develop to successfully meet the demands of their particular sports^1^, and is often related to skills adapted in the childhood^2^. These skills may include, for example, reactions to predominantly visual stimuli, jumping ability that depends on explosive strength and coordination, as well as more complex coordination skills that enable athletes to perform multiple movements simultaneously while continuously processing interoceptive and exteroceptive stimuli^3^. In such complex tests, multiple individual abilities come into play, which must be performed with high precision under time pressure (gross motor skills and partial movements)^4^.

To assess the current performance level of athletes or their course, a variety of sport-specific motor tests have been developed such as condition- and fitness tests, coordination tests, or development tests for children or adults^5^. These tests not only help trainers monitor progress but also enable athletes to evaluate the effectiveness of new training strategies by revealing athletes’ weaknesses. Some of these tests focus on measuring a single skill or ability such as standing long jump or single-leg stand, while others assess multiple abilities simultaneously, offering a more comprehensive view of an athlete’s performance. Examples of the latter include the Deutscher Motorik-Test^6^ and the Eurofit-Test^7^. In this context, it is essential to note that isolating a single ability within a movement task can be quite challenging. All sports-related movements involve the activation of multiple joints and muscles across various degrees of freedom, making it difficult to pinpoint just one specific ability with one test. Moreover, athletes often need to execute multiple skills and abilities simultaneously, aggravating it to attribute their performance to the failure of just one skill.

Implementing these tests often requires significant equipment and manual effort, and they may lack automated evaluation. Additionally, some tests are critically scrutinized and need improvement to ensure they provide reliable and valid data. Virtual reality (VR) offers a promising solution to these challenges by simulating sports-specific stimuli and providing valuable data on an athlete’s performance. However, there are currently only a limited number of official sports motoric tests in VR that adequately evaluate athletes’ performance levels, as most existing tools primarily focus on recovery^8^ or learning purpose^9–11^. To assess reaction ability, a previous study developed a virtual drop-bar test in which martial arts athletes’ reaction times were measured by pressing a controller button^12^. The authors advocate the usage of VR to test psychomotor abilities. For assessing jumping ability, we did not find tools designed to measure an athlete’s actual performance; instead the focus on existing tools was primarily on using VR to train jumping skills^13^. We also found no literature on the use of obstacle courses involving various challenges that require completing multiple motor tasks to assess complex motor skills–when parkour appears, it often serves as a means of exploration or as a form of play, where the focus was less on actual physical movement^14^.

To address this gap, we developed a prototype of a sport-motor test battery that measures reaction time (both motor and sensory), jump height, and the execution of complex tasks involving multiple coordinated movements. To ensure the reliability and validity of these tests, we compare the results with traditional assessments in the real environment (RE). Previous studies have already demonstrated that established reaction tests can be successfully replicated in VR^15^, also for simple and complex ones^12^, leading us to anticipate reliable data outcomes from our VR tool at least for the reaction test. However, to address the limitations of RE conditions, we extended the VR testing scenario compared to RE. While we will approach comparisons with caution in some instances, it is essential to validate the data for plausibility by comparing the performances in VR to those from RE. Since we are interested in developing tests that accurately assess each athlete’s current performance level, we selected non-sport-specific scenarios based on the real tests. Previous findings have shown that performing in a non-specific standardized scene reduces or eliminates the learning effects^16^ that may arise during the assessment process.

## Results

### Test-retest reliability

First, the reliability of each test across both conditions (RE, VR) and at each measurement time point (Pre, Post) are presented in Table 1. High reliability has generally found for each test, along with strong correlations between RE and VR.

**Table 1:** Overview of the descriptive statistics and reliabilities for each test separately.

Test	Param.	ConD.	Pre (M + SD)	Post (M + SD)	ICC	CorR. between conditions	
						RE	VR/VR-W
Drop-bar	Resp. time	RE	0.19 ± 0.01	0.19 ± 0.02	0.858	Pretest: *r* = .445* Posttest: *r* = .416*	
		VR	0.38 ± 0.02	0.38 ± 0.03	0.946		
	React. Time	VR	0.34 ± 0.02	0.34 ± 0.03	0.862	N/A	
	Mov. time	VR	0.04 ± 0.02	0.04 ± 0.02	0.907	N/A	
Jump & Reach	Max. height	VR	2.72 ± 0.19	2.71 ± 0.15	0.836	N/A	
	Abs. height	RE	0.42 ± 0.09	0.40 ± 0.07	0.944	Pretest: *r* = .838** Posttest: *r* = .772**	
		VR	0.42 ± 0.07	0.41 ± 0.07	0.886		
Parkour	Bar T	RE	2.55 ± 0.27	2.60 ± 0.24	0.865	Pretest: *r* = .716** Posttest: *r* = .815**	
		VR	2.55 ± 0.37	2.39 ± 0.35	0.831		
		VR-W	2.33 ± 0.21	2.39 ± 0.37	0.761	Pretest: *r* = .454 Posttest: *r* = .210	
	Ball T	RE	3.04 ± 0.32	2.86 ± 0.36	0.880	Pretest: *r* = .406 Posttest: *r* = .718**	
		VR	3.48 ± 0.59	3.01 ± 0.36	0.519		
		VR-W	2.97 ± 0.64	2.90 ± 0.31	0.512	Pretest: *r* = .045 Posttest: *r* = .839**	
	Box T	RE	1.70 ± 0.23	1.65 ± 0.23	0.925	Pretest: *r* = .442 Posttest: *r* = .884**	
		VR	2.18 ± 0.35	1.96 ± 0.27	0.723		
		VR-W	2.00 ± 0.26	1.89 ± 0.28	0.756	Pretest: *r* = .700** Posttest: *r* = .772**	
	Movement T	RE	7.23 ± 0.68	7.11 ± 0.72	0.943	Pretest: *r* = .743** Posttest: *r* = .901**	
		VR	8.21 ± 1.07	7.36 ± 0.87	0.684		
		VR-W	7.30 ± 0.91	7.18 ± 0.73	0.845	Pretest: *r* = .566* Posttest: *r* = .794**	

Reliability is presented using the intraclass correlation coefficient (ICC) for both pre- and post-measurements, as well as between the conditions, shown in the right column. N/A indicates that these measurements could not be measured in RE. To compare the conditions, Pearson’ r was used to calculate the correlations, with thresholds defined as follows: 0.1–0.3 small, 0.3–0.5 moderate and > 0.5 large correlation. Significance levels are denoted as follows: *p* < .05 (*), and *p* < .01(**). VR-W stands for virtual reality with opponent. The units specified for the tests are as follows: seconds for the drop-bar and parkour test, meters for the jump and reach test.

### Comparison with RE – tests for differences

Secondly, the difference tests are illustrated in Table 2. The factor time and the interactions between time and condition were not significant. There were significant differences between the conditions for the drop-bar and parkour test.

**Table 2:** The results of the MANOVA/ANOVA for each test. The factor time includes pre- and posttest, the factor condition RE and VR. In the parkour test, the condition virtual reality with opponent (VR-W) was also included in the analysis. The number of participants who completed the tests is represented by n.

Factor	Significance	Effect
Drop-bar		
Two-factor ANOVA with repeated measurements and the calculated effect sizes for the dependent variable: Response Time (*n* = 26)		
Condition	*F*(1,25) = 1019.989, *p* < .001, partial η² = 0.976, Wilks-Lambda: 0.024	large
Time	*F*(1, 25) = 1.022, *p* = .322, partial η² = 0.039, Wilks-Lambda: 0.961	no significant effect
Condition*time	*F*(1, 25) = 0.502, *p* = .485, partial η² = 0.020, Wilks-Lambda: 0.980	no significant effect
Post-hoc: The differences between the conditions occurred both in the pre- and post-test (*p* < .01).		
Jump and reach		
Two-factor ANOVA with repeated measurements and calculated effect sizes for the dependent variable: Absolut Jump Height (*n* = 25)		
Condition	*F*(1, 24) = 0.615, *p* = .440, partial η² = 0.025, Wilks-Lambda: 0.975	no significant effect
Time	*F*(1, 24) = 1.005, *p* = .326, partial η² = 0.040, Wilks-Lambda: 0.960	no significant effect
Condition*time	*F*(1, 24) = 1.110, *p* = .303, partial η² = 0.044, Wilks-Lambda: 0.956	no significant effect
Parkour		
Two-factor MANOVA with repeated measurements and the calculated effect sizes for the dependent variables: Movement time, Bar time, Ball time, Box time, Bar hits, box hits		
Condition	*F*(2, 26) = 12.362, *p* < .001, partial η² = 0.738, Wilks-Lambda: 0.069	large effect
Time	*F*(2, 26) = 2.547, *p* = .106, partial η² = 0.586, Wilks-Lambda: 0.414	no significant effect
Time*condition	*F*(2, 26) = 1.932, *p* = .066, partial η² = 0.305, Wilks-Lambda: 0.483	no significant effect
The ANOVAs indicate differences between the conditions in all dependent variables (*p* < .05). Post-hoc comparisons:		
Movement time	In VR, the participants needed significant longer (*p* < .001) to complete the parkour compared to RE (*M*_Diff_ = 0.449, 95%-CI[0.135, 0.764]) and VR-W (*M*_Diff_ = 0.581, 95%-CI[0.213, 0.949]).	
Bar time	In RE, the participants needed significant longer (*p* < .05) to complete the slalom compared to VR (*M*_Diff_ = 0.142, 95%-CI[0.030, 0.254]) and VR-W (*M*_Diff_ = 0.259, 95%-CI[0.054, 0.464]).	
Ball time	In VR, the participants needed significant longer (*p =* .003) to complete the ball task compared to VR-W (*M*_Diff_ = 0.335, 95%-CI[0.117, 0.552]).	
Box time	In RE, the participants were significant faster (*p* < .001) to complete the jumps over the boxes compared to VR (*M*_Diff_ = -0.364, 95%-CI[-0.507, -0.222]) and to VR-W (*M*_Diff_ = -0.235, 95%-CI[-0.355, -0.116]). In VR-W, the participants were also faster (*p* = .016) compared to VR (*M*_Diff_ = -0.129, 95%-CI[-0.235, -0.023]).	
Hit bars	In RE, significant more hits (*p* = .002) were detected compared to VR (*M*_Diff_ = 0.333, 95%-CI[0.130, 0.537]). No significant differences between VR and VR-W (*p* > .05).	
Hit boxes	In RE, significant less hits (*p* < .001) were detected compared to VR (*M*_Diff_ = -0.726, 95%-CI[-1.081, -0.372]) and compared to VR-W (*M*_Diff_ = -0.548, 95%-CI[-0.875, -0.221]). No significant differences between VR and VR-W (*p* > .05).	

Further statistical analyses were conducted separately for each test to determine possible differences between pre- and post-test for each condition (RE, VR).

For the Drop-Bar, no significant differences between the pre-and post-test in VR for response time (*p* = .267), reaction time (*p* = .514), movement time (*p* = .636) and in RE for response time (*p* = .754). For the Jump and Reach in VR, no significant differences occurred between the pre-test and post-test, either for the absolute heights or the maximum heights (*F*(1, 24) = 0.165, *p* = .849, partial η² = 0.014, Wilks-Lambda: 0.986). This also applies for the absolute heights in RE (*p* > .05). In the parkour test, there are significant differences within the time for the RE (*F*(1, 25) = 10.494, *p* < .001, partial η² = 0.656, Wilks-Lambda: 0.344). This applies to the movement time (*F*(1, 25) = 7.327, *p* = .012, partial η² = 0.227), the ball time (*F*(1, 25) = 22.434, *p* < .001, partial η² = 0.473), the box time (*F*(1, 25) = 4.529, *p* = .043, partial η² = 0.153). Lower values were recorded in the post-test for the majority of the calculated times. There are significant differences within the time for the VR (*F*(1, 25) = 424.992, *p* < .001, partial η² = 0.988, Wilks-Lambda: 0.012). This applies to the movement time (*F*(1, 25) = 33.086, *p* < .001, partial η² = 0.580), the bar time (*F*(1, 25) = 11.226, *p* = .003, partial η² = 0.319), the ball time (*F*(1, 25) = 24.345, *p* < .001, partial η² = 0.504), the box time (*F*(1, 25) = 19.415, *p* < .001, partial η² = 0.447). Lower values were recorded in the posttest for the majority of the calculated times. There are no significant differences within the time for the VR-W (*F*(1, 18) = 1.175, *p* = .361, partial η² = 0.239).

The number of hits in the parkour test shows differences between RE and VR conditions. In both VR conditions (VR, VR-W), no hits were detected in the bar slalom, neither in the pre- nor the post-test. For the boxes, an average of 0.57 (± 0.4) was recorded in the pre-test and 0.52 (± 0.57) in the post-test. In RE, the results were opposite, with zero hits on the boxes, but 0.26 (± 0.29) in the pre-test and 0.40 (± 0.46) in the post-test.

## Discussion

In the current study, three virtual reality tests were developed to assess athletes’ performance in reaction ability (drop-bar test), jump ability (jump and reach test, including the lower extremity strength ability), and the detection of speed (time needed to complete) and accuracy (hits with the objects) for a variety of complex movement patterns (parkour test). These tests were based on similar assessments performed in real environmental conditions to ensure comparability. Since VR offers advantages that cannot be replicated in real environment, some modifications were made, particularly in the reaction test. Before recommending these tests or developing additional ones, it is essential to establish their validity and reliability first. To accomplish this, we analyzed the data from each test separately under each condition. For validity, we also compared the results between RE and VR.

In terms of reliability, the data indicate high reliability from pre- to post-test for both the drop-bar and the jump and reach test across all conditions, aligning with previous studies–particularly for reaction ability^12,15,17^. These studies demonstrate that these tests fall within a range of effective reaction assessments and confirm the suitability of VR devices for this purpose. This is further supported by the good to excellent (mostly excellent) reliability (indicated by high ICCs) for all parameters within the drop-bar and jump and reach test. Although ICCs of similar magnitude were observed in the parkour test, there were greater deviations from the pre- to the post-test in the tasks involving picking up the ball and placing it on the storage area in VR. This may because, in VR, picking up the ball requires only touching it with the virtual hand, after which the ball automatically follows the hand’s position. Once the ball touches the storage place, it remains there, which differs from RE and where it may take time to adjust to^18^. Although these movements may appear easier in VR, observations indicated that the test subjects felt more insecure in this condition. They tended to linger visually longer in the target position, as the movement itself was unfamiliar, and they did not receive any haptic feedback to confirm whether the ball was resting on the surface.

The times for this task in the posttest were significantly lower than in the pretest, likely indicating a learning effect that influenced the reliability of the data. Good reliability was demonstrated not only through high ICCs but also by the subsequent tests for significant differences in the time factor [pretest, posttest], which further support these findings.

To assess validity, differences and correlations were analyzed to interpret the results. In the drop-bar test, significant differences were observed between the RE and VR. This outcome was expected, as the motor tasks differed substantially between the two conditions (*p* < .05): in RE, participants completed the task by simply closing their hand, whereas in VR, they had to move the controller from the right to the left side until it made contact with the virtual bar. Strong positive correlations were found between the response time in RE and VR (the response time was the most comparable parameter), indicating that participants who reacted faster in RE also tended to react faster in VR, that is in line with the outcome of other studies^12^. This finding supports high construct validity. Tracking sensory reaction time (referred to here as reaction time) and the movement time could provide valuable insights for future analyses, particularly in studies focused on movement patterns that more closely resemble real-life scenarios. This approach would allow for a more precise differentiation between groups, such as athletic vs. non-athletic individuals, or novices vs. experts, enabling a clearer understanding of performance differences across skill levels^19^. To further validate the reaction ability tool, it should be incorporated into intervention studies focused on training reactions–often associated into developing of anticipatory skills^20^. This will allow for the assessment of both the starting and endpoint performance levels of participants.

The jump and reach test also demonstrated high validity, as no significant differences and strong correlations were observed between RE and VR. The technical setup–using the controller to measure baseline and maximum reached height–proved to be a precise method for assessing participants’ jump ability, with differences between RE and VR of less than 1 cm. Hereby, lighter and even more precise devices, such as the Oculus Quest 2^21^ (newer ones are available), could further enhance data validity compared to the currently used ones. This procedure could be applied to other jumping tests to expand the testing domain of the VR test battery such as the Counter Movement Jump or others^22^.

Reduced validity was observed when comparing RE and VR for the parkour, as participants required approximately one additional second to complete it in VR. The dimensions and their arrangement of the virtual objects were identical to those of real environment, which had been previously scanned, similar to methods used before^23^. The participants might experience insecurity in VR, although naturally walking is the most acceptable locomotion technique compared to others^24^. Therefore, it is essential to let them explore the virtual environment first before they engage with the task to eliminate this factor. However, when a virtual opponent was introduced into the scene, the initial insecurity appeared to diminish; the completion times between RE and VR-W no longer showed significant differences (*p* > .05). This suggests that the presence of the opponent positively influences participant behavior and can be regarded as a source of motivational support that is in line with other studies, especially when the opponent outperformed the user^25^. However, it has also been demonstrated that the inclusion of a virtual opponent does not always have a positive impact on participants^26^. Therefore, it is essential to consider the context of the specific task being performed. Due to its lower reliability in the ball task, the parkour test can also be seen as a “training session” than a strict “test”. While it may be beneficial for practice, certain motor tasks–such as picking the ball–cannot be fully replicated due to the absence of haptic feedback, which significantly limits current VR applications. By adding vibrotactile or force feedback, the degree of presence and performances increase, especially when interacting with objects^18^. To maintain comparability between the VR and RE condition, the VR-W was always conducted after VR. This may have affected the results, as habituation to the VR running scenario could have occurred.

Overall, the feedback questionnaire indicate that participants found the tests highly enjoyable and were highly motivated to complete them. Research has shown that tasks involving jumping skills in VR enhance motivation and the sense of presence among participants^27^. However, some participants expressed discomfort, particularly during the tasks that required jumping while wearing the head-mounted display. Despite being securely fastened, the weight of the head-mounted display and the potential (however minimal) for it to shift or fall created a perceived limitation for some participants. Nevertheless, the jump heights did not differ significantly between the RE and VR. However, there were noticeable outliers in the VR data compared to the RE data, which should be considered when interpreting the suitability of VR for sports applications or assessments. This suggests a greater level of insecurity or measurement errors during the initial phases, highlighting the importance of incorporating acclimatization trials. The insecurity is also evident in the number of hits. Significant differences were observed in the frequency of hits during the slalom, with more hits occurring in RE compared to VR. In RE, the bars were hit more frequently, possibly due to the lack of the full-body visualization and detection in VR setup. In VR, only the feet could collide with the bars, whereas in RE, the whole body could make contact. Conversely, the impact with the boxes was different: in RE, hitting the boxes resulted in a stronger impact, while in VR, the absence of physical resistance made it easier to accept the hits.

In summary, VR serves as a practical tool for assessing the parameters examined in this study. The current VR prototype offers three types of tests–reaction time, jumping ability, and speed and accuracy of complex movement patterns–that can be selected and completed sequentially, eliminating the need for physical equipment. As more data is collected, performance metrics can be categorized by demographic factors such as age, gender, and sport type. Z-transferred values can be used to determine the athlete’s weaknesses. However, it would be utopic to claim that VR can quantify all known motoric abilities, as the absence of haptic feedback limits its capacity to accurately simulate a range of tasks. Although this has been noted for some time^28^, addressing this limitation should be a priority in future VR development efforts.

## Methods

### Participants

In total, 32 participants (9 females, 23 males, 25.54 ± 3.71 years) were collected and went through the pre-and post-tests, and the interventions. Before starting the experiment, the participants were demanded to fill out a self-created questionnaire in which pre-experiences with the sport-motor tests exist. This also concerns VR pre-experiences that could influence participants’ performances in the VR scenario itself. The participants had less experience in terms of VR experience, as most had only taken university courses or participated in VR studies. The virtual opponent was generally perceived as realistic, although some participants did not notice it because they were too focused on completing the motor tasks within the course. 50% of the participants reported wearing visual aids, primarily contact lenses. 53% had their dominant eye on the right side, while 28% had no prior experience participating in VR studies. Additionally, 5% of the participants had no experience with gaming. On average, the participants had engaged with at least two types of video games, including strategy, racing, first-person shooters, role-playing games, and music or rhythm games. Furthermore, 66% of the participants were right-handed. On average, participants spent 22 h per week engaging in sports and had been active for 14 years. They also played video games for an average of 4.5 h per week. Regarding attitudes toward technology, participants rated their openness to new technology at 4.26 on a Likert scale (with 1 being “not applicable” and 5 being “applicable”). They expressed a neutral stance on the potential use of VR technology in daily activities, scoring it 3.52, but were more open to VR in sports (4.17) and research (4.57).

After the study, the participants found task performance in VR enjoyable (4.58), considered the VR experience realistic (4.08), and reported moderate experience with virtual opponents (3.46). They rated their ability to handle the VR tasks as moderate (2.49). The participants came from a wide range of sports, showcasing remarkable diversity. These included frisbee, racing cycling, strength training, soccer, Thai boxing, swimming, table tennis, track and field athletics, couple dancing, climbing, pole fitness, flag football, and rowing. Approximately 75% of the athletes participated in individual sports, while the remaining 25% were involved in team sports, which often demand quick reaction times, jumping ability, or complex motor coordination.

Power calculations to detect the numbers of participants were performed using G*Power software (version 3.1).

### Procedure

**Fig. 1:**
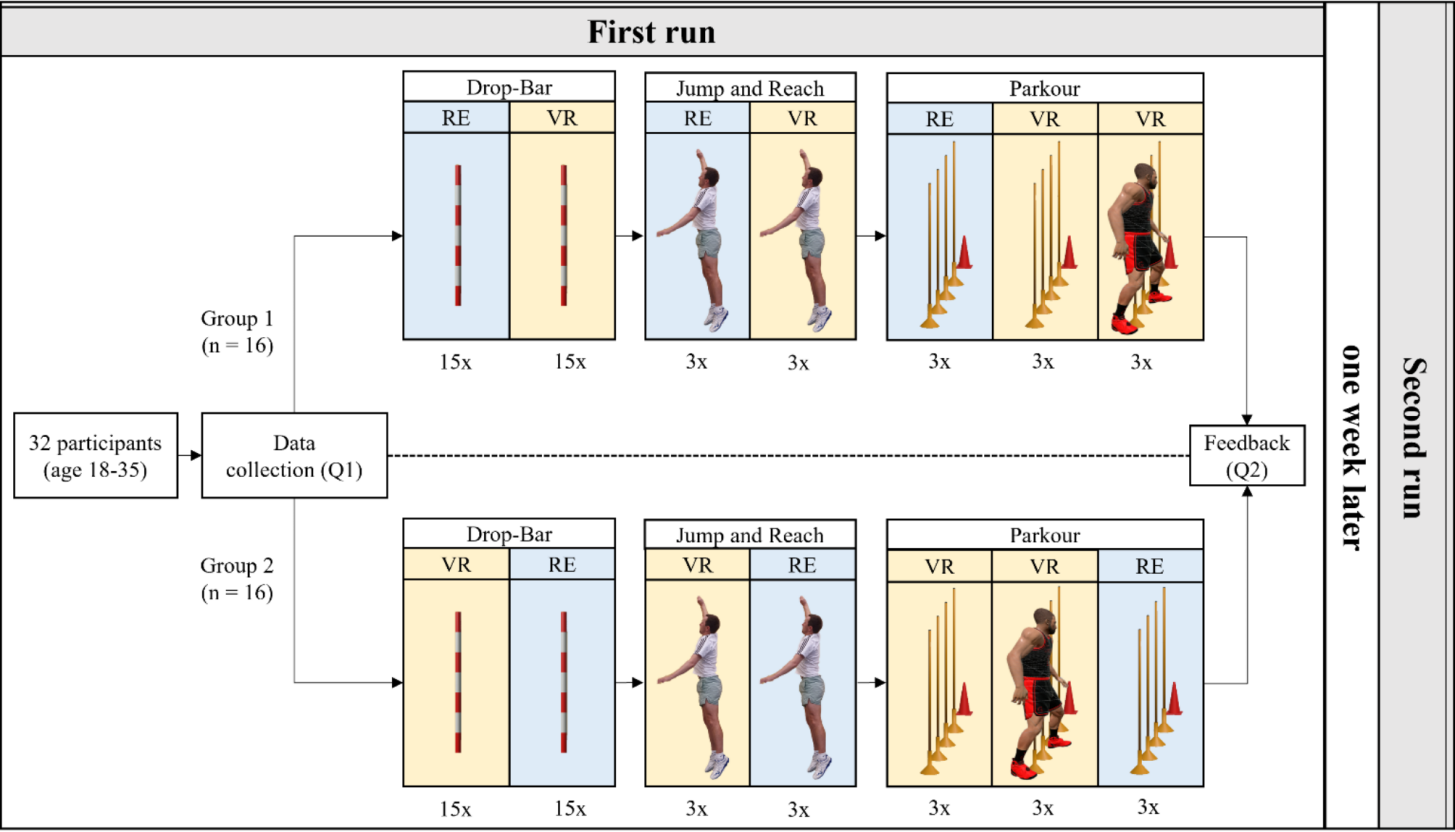
Overview of the Study design. The participants began with either the VR or RE condition. One week later, they completed the same tests, with the order reversed from the previous week.

The following figure shows the procedure of the current study (see Fig. 1). Before the experiment began, participants were asked to complete a custom-designed questionnaire (Q1). This survey gathered information about their prior experiences with VR and sports, demographic details and their attitude towards fusing new technology in sports research. All participants received the instructions prior to the study and gave their written informed consent. The study was approved by the authors’ university ethics committee, and all procedures were conducted in accordance with the committee’s guidelines and regulations. In addition, written informed consent was obtained from all subjects and/or their legal guardian(s) for the publication of identifying information and/or images in this online open-access publication.

Generally, the participants completed all three tests twice, once in RE and VR. Depending on their group assignment, they either began with RE or VR, and one week, they switched to the alternative condition. The participants were pleased to complete test trials first (in both conditions) to ensure comprehension and awareness of VR’s interaction possibilities. The Simulator Sickness Questionnaire (SSQ)^29^ was handed out before and after the VR tasks. Since no significant symptoms, such as nausea, disorientation or oculomotor issues, were reported, we deemed the tasks to be feasible.

#### Drop-bar

##### RE

**Fig. 2:**
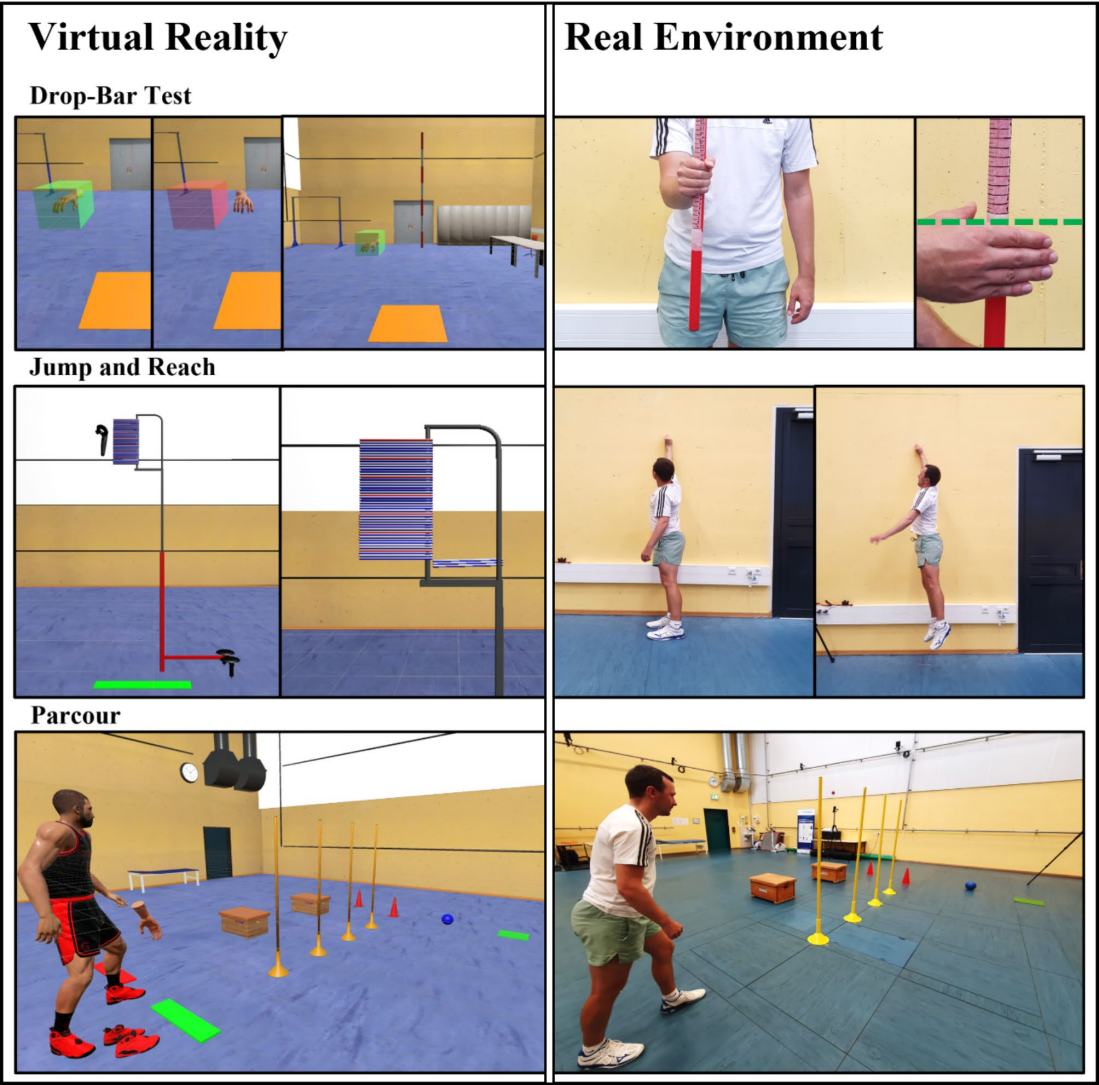
The illustration of each test conducted in VR (left column) and real environment (right column). The green dashed line in the Drop-Bar Test real environment (right column) indicates the baseline from which the measurements began in the real environment. In the jump-and-reach test in VR, participants touched virtual panels with the controller; the panels would then swing upon contact. In real environment, participants used chalk to mark their maximum jump height directly on the wall. In the parkour test, the final three trials included the presence of a virtual opponent.

In this test, participants were instructed to stand in front of the experimenter (Exp) with their right arm extended. The Exp then placed a bar between the participant’s hand. After a random interval, the Exp released the bar, and the participants were required to catch it by closing their fingers (Fig. 2, upper row right). The participants were required to stand while performing the task and instructed to hold their arm in a position that matched the posture used in VR. This was done to ensure comparable body posture across conditions. The test ended after 15 trials were completed. We extended the number of trials (typically up to 5^30^) to further analyze whether habituation to the task demands occurred.

##### VR

In this test, participants stood on an orange square marked on the ground, with a transparent green cube positioned to their front right (Fig. 2, upper row left). They were given a controller, represented by a hand-mesh on the screen. When they moved their hand into the cube, it would turn green, signaling the start of the experiment. A bar then appeared at a randomized interval in front of them and began to fall at a uniformly increasing speed. If the participant’s hand left the cube, it turned red. When the trial was finished, they needed to move their hand back to the red square; otherwise, the next trial could not be initiated, preventing them from cheating. Upon reaching the bar, a sound provided feedback on whether they had successfully intercepted it. The arm moved from right to left so that the hand mesh only needed to touch the bar, rather than gripping it as would be required in the RE. The next trial began once they returned to the cube. The test ended after 15 trials were completed. Care was constantly taken to ensure that participants were permitted to use their dominant hand at all times.

#### Jump and reach

##### RE

The participants were instructed to stand along a line parallel to the wall (30 cm), positioning their shoulder of the dominant side toward it. They held a piece of chalk between their thumb and index finger. First, they stretched upward to mark their maximum reaching height on the wall by drawing a line. Following this, they performed three jumps, touching the wall with the chalk at the peak of each jump (Fig. 2, second row). The chalk was held between the index and middle finger, similar to how the controller was held in VR. This was done to maintain a comparable reach height across conditions, ensuring reliability in the experimental process.

##### VR

The participants were instructed to stand on the green line, positioning their right shoulder forward. They held the controller in their right hand. To establish a baseline, they were asked to pull the controller as high as possible without lifting onto their toes. This movement was used for calibration, allowing the system to calculate each participant’s jump height accurately. Once the calibration was completed, the experimenter gave the starting signal. Participants were required to complete three jumps in total, with sufficient rest time provided between each trial. To boost motivation, we incorporated interactive panels that rotate when touched with the controller (Fig. 2, second row). To check a reliable test execution, we also recorded the maximum reached height in both RE and VR. No significant differences were observed between the conditions (*F*(1, 22) = 0.003, *p* = .956, partial η² = <0.001, no effect) or in the interaction (*F*(1, 22) = 0.496, *p* = .489, partial η² = 0.022, no effect). This demonstrates consistent reached height acquisition throughout the experiment.

#### Parcour

##### RE

The parkour course consisted of 4 bars, 2 pylons, a ball, a storage area, and 2 boxes. Participants were required to complete the course 3 times as quickly as possible. To ensure consistency in movement, they were instructed not to touch any objects. First, they had to navigate through the bars in a slalom pattern. After passing the bars, they were to pick up the ball and place it in the storage area (Fig. 2, third row). Finally, they had to jump over the 2 boxes and continue straight to the finish line. Between trials, participants took breaks to recover, helping to prevent fatigue across sessions.

##### VR

The same procedure was followed in the VR condition, with the exception that 6 trials were conducted. In the final 3 trials, a virtual opponent appeared (see Fig. 2), running through the parkour at a fast pace (the opponent completed the parkour in 6.10 s). Additionally, collider detection for the participants’ feet was enabled to track any collisions with the virtual objects. Although participants did not experience any haptic resistance due to the removal of the real objects for safety reasons, these collisions were still recorded. To primary ensure a valid comparison between RE and VR, the first three runs were conducted without opponent, as it was the case in RE. Once the opponent was introduced, further analysis was carried out to determine whether the presence of the opponent positively influenced participants’ performances.

### Data analysis and statistics

Before detailing the individual processes for each test, the fundamental procedures were outlined. Initially, the normal distribution of each data row was assessed using the Shapiro-Wilks test^31^. Additionally, each dataset was scrutinized using the Median Absolute Deviation (MAD) score, a robust method compared to other filtering techniques^32^. We also carefully examined the data for variance homogeneity.

To ensure data reliability, we calculated the intraclass correlation coefficient (ICC) for each dependent variable at both the first measurement point (pretest) and the second (posttest), assessing each test separately. The ICC can be categorized as follows: poor reliability < 0.75, good 0.75 to 0.9, and excellent reliability > 0.9^33^.

**Fig. 3:**
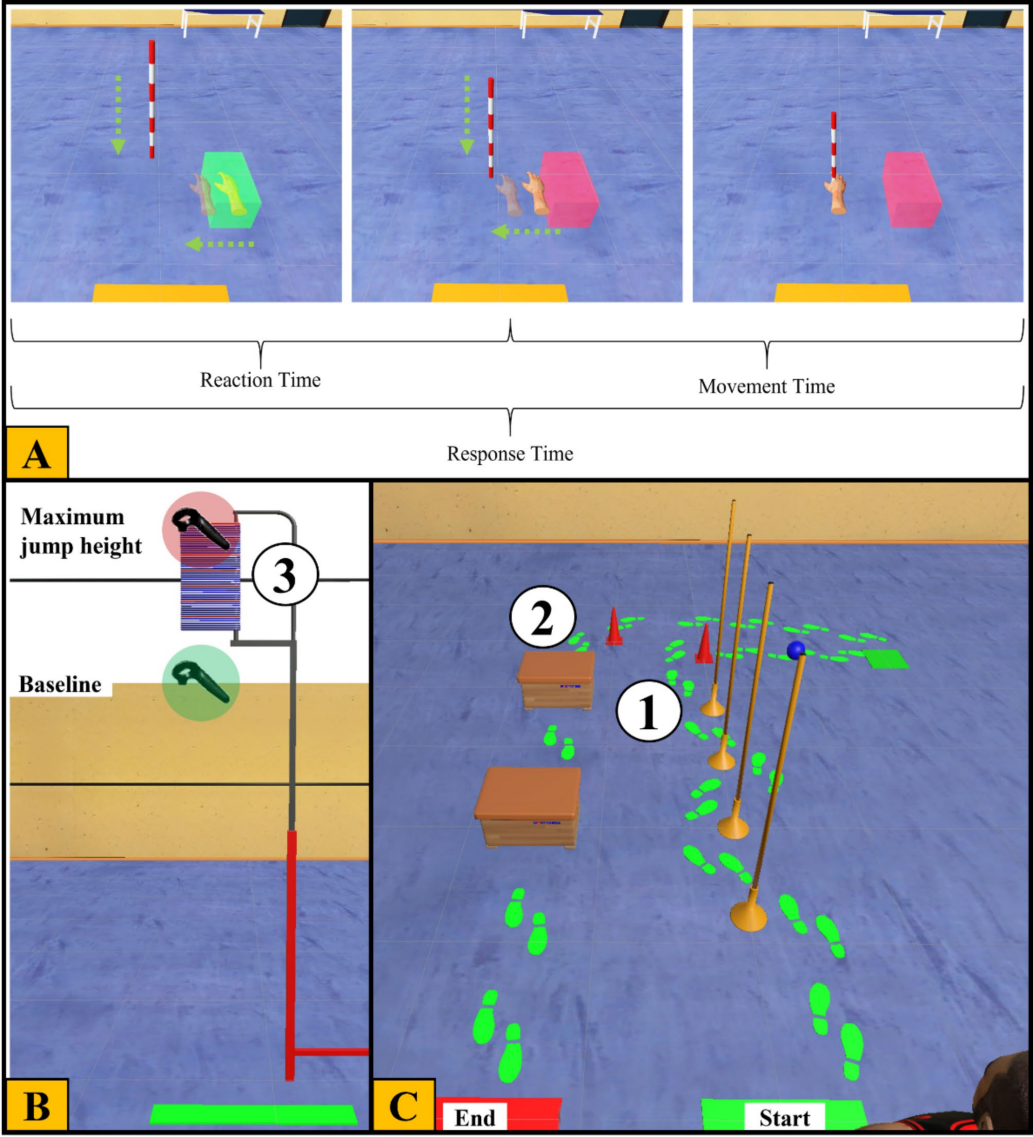
The determination of the parameters. The Drop-Bar Test is depicted in (**A**), showing the segmented time intervals. In (**B**), the height calculations for the Jump and Reach Test are illustrated. The controller, highlighted by a green transparent square, indicates the baseline, representing the participant’s maximum reach. Both, the baseline and the panels (3) are calibrated accordingly. At the peak of the jump, the maximum height is captured, represented by the controller with the red transparent square. The absolute jump height was calculated by subtracting the baseline from the maximum jump height. In (**C**), the parkour course and the path that participants were required to follow (green footprints). The bar time was measured from the start line to 1, the ball time from 1 to 2, and the box time from 2 to the end line. The movement time is the sum of all times together.

For validation, a 2 × 2 multifactorial analysis of variance (MANOVA) with repeated measures was employed, with the within-subject factors being time [pre, post] and condition [RE, VR] for each test separately, if more than one dependent variable was included. This statistical analysis was chosen to determine whether a significant interaction goes along with the measurements between RE and VR. For each test, the dependent variables were: three measures for the Drop-Bar Test in VR [Reaction Time, Movement Time, Response Time] and one measure in RE [Response Time], two for the Jump and Reach Test in VR [absolute height, maximum height] and one in RE [absolute height], and six for the Parkour Test [movement time, bar time, ball time, box time, hit bars, hit boxes]. A detailed description of the recorded times is given in Fig. 3. Bonferroni-corrected post-hoc comparisons were performed only when the main effects reached statistical significance (*p* < .05). When only one dependent variable was comparable, we used a 2 × 2 ANOVA instead. Only the parameters present in both conditions were considered to analyze the differences between RE and VR. Thus, the comparison within the Drop-Bar Test was evaluated by analyzing Response Time, while the Jump and Reach Test was assessed using the absolute height measurements. For the Parkour Test, all previously described parameters were analyzed. In VR, these parameters were measured under normal conditions and with the appearance of an opponent.

The tests between pre- and post-test aimed to identify learning effects and, consequently, lower reliability, whereas the tests considered the comparison between the RE and VR conditions indicating lower validity if significant differences arose. To assess the construct validity of the data recorded in the VR condition, Pearson’s correlation coefficient was calculated separately for the dependent variables between the VR and RE conditions, where 0.1 to 0.3 stands for a small, 0.3 to 0.5 a moderate, and > 0.5 a large effect.

The calculation and determination of the dependent variables are illustrated and explained in Fig. 3. In the Drop-Bar test within the RE, reaction time was calculated based on the distance the hand traveled from a predetermined baseline. The calculation (t = $\:\sqrt{2s/g}$) follows the guidelines of the official protocol from the Institut für Angewandte Trainingswissenschaft (IAT, Institute of Applied Training Science). Figure 3 (A) illustrates the calculation of the reaction time in VR. In the Jump and Reach test, the jump height was also calculated by subtracting the baseline from the maximum jump height, similar to the method used in VR (see Fig. 3B). The path of the Parkour test and corresponding time intervals, which were the same for RE and VR, are visualized in Fig. 3 (C).

### Experimental apparatus

#### Hardware

The virtual environment was presented using the HTC Vive Pro Eye (HTC, Taiwan), which offers a 110° field of view. A wireless adapter was installed to enable unrestricted movement within a 50-square-meter area. Four base stations were strategically positioned in the laboratory to cover the manufacturer-specified tracking area of 100 square meters. Each eye’s display resolution of 1440 × 1600 ensured a clear and sharp visual experience, minimizing pixelation. For task interactions, participants used the 2018 HTC Vive Controller, equipped with 24 sensors for precise tracking (HTC, Taiwan). Additionally, two HTC Vive trackers 3.0 were attached to the participants’ ankles, securely fixed in place using straps. They were used for real-time foot visualization, enabling the detection of any contact with obstacles (non-haptic). The HTC VIVE headset weighs 555 g, one controller weighs 203 g, the wireless adapter weighs 370 g, and the two trackers together 182 g. The total weight of all components is 1310 g.

The system ran on a high-performance setup, featuring an Intel i7 CPU, 16 GB of RAM, a 512 GB SSD, and an NVIDIA GTX 1080 graphics card with 8 GB of RAM. A dongle extension was utilized to manage the connectivity for all VR system components, including base stations, controllers, and trackers.

#### Software

The virtual environment was developed using Blender (version 3.1.2), where each virtual asset was modeled and textured. To ensure accuracy, original dimensions were measured adhering to standard specifications. The completed scene was then imported into Unity (version 2021.3.16f1), with SteamVR (version 1.26) integrated to enable VR interactions. Scene functionality was implemented using C# scripts.

The humanoid avatar, developed by Code This Lab (Italy, 2009), was sourced from the Unity Asset Store. All the parkour animations needed were self-recorded using Vicon Shogun (version 1.6.3) and applied to the avatar’s skeleton.

Data processing and visualization were carried out in Python (version 3.10.8), while further statistical analysis was performed using SPSS (version 29).

## Data Availability

Data is provided within the manuscript or supplementary information files.
